# Release of a liver anticancer drug, sorafenib from its PVA/LDH- and PEG/LDH-coated iron oxide nanoparticles for drug delivery applications

**DOI:** 10.1038/s41598-020-76504-5

**Published:** 2020-12-09

**Authors:** Mona Ebadi, Saifullah Bullo, Kalaivani Buskara, Mohd Zobir Hussein, Sharida Fakurazi, Giorgia Pastorin

**Affiliations:** 1 Materials Synthesis and Characterization Laboratory, Institute of Advanced Technology (ITMA), Universiti Putra Malaysia, Selangor, Malaysia; 2 Department of Linguistic and Human Sciences, Begum Nusrat Bhutto Women University, Sukkur, Sindh, 65200 Pakistan; 3 Department of Human Anatomy, Faculty of Medicine and Health Sciences, Universiti Putra Malaysia, 43400, Serdang, Selangor, Malaysia; 4 Laboratory of Vaccine and Immunotherapeutics, Institute of Bioscience, Universiti Putra Malaysia, 43400, Serdang, Selangor, Malaysia; 5 Department of Pharmacy, National University of Singapore, Singapore, Singapore

**Keywords:** Biochemistry, Biotechnology, Cancer, Cell biology, Chemical biology, Developmental biology, Drug discovery, Medical research, Chemistry, Materials science, Nanoscience and technology

## Abstract

The use of nanocarriers composed of polyethylene glycol- and polyvinyl alcohol-coated vesicles encapsulating active molecules in place of conventional chemotherapy drugs can reduce many of the chemotherapy-associated challenges because of the increased drug concentration at the diseased area in the body. The present study investigated the structure and magnetic properties of iron oxide nanoparticles in the presence of polyvinyl alcohol and polyethylene glycol as the basic surface coating agents. We used superparamagnetic iron oxide nanoparticles (FNPs) as the core and studied their effectiveness when two polymers, namely polyvinyl alcohol (PVA) and polyethylene glycol (PEG), were used as the coating agents together with magnesium–aluminum-layered double hydroxide (MLDH) as the nanocarrier. In addition, the anticancer drug sorafenib (SO), was loaded on MLDH and coated onto the surface of the nanoparticles, to best exploit this nano-drug delivery system for biomedical applications. Samples were prepared by the co-precipitation method, and the resulting formation of the nanoparticles was confirmed by X-ray, FTIR, TEM, SEM, DLS, HPLC, UV–Vis, TGA and VSM. The X-ray diffraction results indicated that all the as-synthesized samples contained highly crystalline and pure Fe_3_O_4_. Transmission electron microscopy analysis showed that the shape of FPEGSO-MLDH nanoparticles was generally spherical, with a mean diameter of 17 nm, compared to 19 nm for FPVASO-MLDH. Fourier transform infrared spectroscopy confirmed the presence of nanocarriers with polymer-coating on the surface of iron oxide nanoparticles and the existence of loaded active drug consisting of sorafenib. Thermogravimetric analyses demonstrated the thermal stability of the nanoparticles, which displayed enhanced anticancer effect after coating. Vibrating sample magnetometer (VSM) curves of both produced samples showed superparamagnetic behavior with the high saturation magnetization of 57 emu/g for FPEGSO-MLDH and 49 emu/g for FPVASO-MLDH. The scanning electron microscopy (SEM) images showed a narrow size distribution of both final samples. The SO drug loading and the release behavior from FPEGSO-MLDH and FPVASO-MLDH were assessed by ultraviolet–visible spectroscopy. This evaluation showed around 85% drug release within 72 h, while 74% of sorafenib was released in phosphate buffer solution at pH 4.8. The release profiles of sorafenib from the two designed samples were found to be sustained according to pseudo-second-order kinetics. The cytotoxicity studies confirmed the anti-cancer activity of the coated nanoparticles loaded with SO against liver cancer cells, HepG2. Conversely, the drug delivery system was less toxic than the pure drug towards fibroblast-type 3T3 cells.

## Introduction

Currently, there are many treatments to minimize cancer cell propagation^1^, such as surgery, chemotherapy and radiation therapy^2,3^. Chemotherapy for cancer treatment has been used from the beginning of the twentieth century^4,5^. Using this approach, cancer cells’ growth can be inhibited by interfering or destroying cellular structures to stop the proliferation of tumor cells. The purpose of chemotherapy is to eliminate cancer cells that are more susceptible to chemotherapy drugs due to their much faster growth and division rates than healthy cells. Unfortunately, therapeutic agents cannot distinguish between tumors and healthy cells, and as a result, chemotherapy also affects healthy cells^6−8^. The effectiveness of a therapeutic approach directly depends on its ability to kill cancer cells in such a way that healthy cells of the body are not affected. The major disadvantage of most chemotherapy drugs is the lack of selectivity, which has potential side effects on healthy tissues and cells. To solve this problem, targeting magnetic medication using the absorption property of magnetic nanoparticle carriers through an external magnetic field is used to increase the delivery of active molecules to specific sites in the body^9−12^.

Among the most recent drug delivery carriers, magnetic nanoparticles have been considered for improving drug release, increasing its availability in the bloodstream and reducing its systemic toxicity^13^. These nanostructures are superior in terms of biocompatibility, as they can protect drug molecules from nonspecific interactions and premature elimination from the body. To achieve this, they need to have a small particle size, can cross biological barriers for delivery of the drug to the target site and thus increasing the drug’s therapeutic efficacy, and possibly reduce the dosage^14−17^. At the same time, the biocompatibility of the magnetic nanoparticles can be enhanced by coating them with polymers such as polyvinyl alcohol, polyethylene glycol, or chitosan.

The non-toxicity, chemical stability and magnetic properties of magnetic-based nanoparticles.

The presence of the external magnetic field is the main reason for its wide application in the pharmaceutical industry. These unique features make them more efficient than other nanostructures. These particles can be applied in different industries, but their role in biomedical applications is particularly significant, especially in the field of drug delivery, owing to their intrinsic magnetism^18−20^.

The optimization of magnetic nanoparticles depends on several factors, particularly their particle size. Particles, after entering the body upon intravenous injection, are quickly recognized by the immune system and the spleen if their size is larger than 200 nm and they are ultimately removed by phagocytosis cells that lead to reduced blood circulation. On the other hand, particles smaller than 10 nm are rapidly refined via kidneys and extravasation. The optimum size for these particles is between 10 and 200 nm, as these nanoparticles are small enough to evade by the reticuloendothelial system (RES), and therefore they can penetrate difficult sites and small capillaries as well as, they can take advantage of the larger fenestrations of blood vessels at the cancer site to extravagates and accumulated where they are most needed^21−24^.

Iron oxide nanoparticles as nanocarriers with various biomedical applications; targeted drug delivery to tumors and cancer treatment. These applications are possible due to their simple separation by the external magnetic field, magnetic properties and capability to carry therapeutic agents. The efficiency of these magnetic nanoparticles is due to their right size with magnetic properties.

Furthermore, recent works on iron oxide nanoparticles have shown that these nanoparticles do not demonstrate in vivo, immediate or long-term toxicity. For this reason, it is anticipated that the targeted and effective therapeutic approaches can be achieved using magnetic nanoparticles that can be used in medicine, where side effects, as well as biological damages resulting from chemotherapies, using this method, could be reduced.

Uncoated magnetic nanoparticles are known to be chemically active and which can be easily oxidized in the air. This generally leads to loss of magnetic properties and dispersion. So for many applications, the development of protective strategies for the stabilization of uncoated magnetic nanoparticles against degradation is very vital. These strategies are including bonding or coating with organic samples, surface stabilizers or polymers^20,25,26^. It is worth noting that in many cases, the protective layer does not only stabilize the nanoparticles, but it can also be used to connect with other nanoparticles or different ligands^27,28^.

Over the past half-century, there have been several advances in the production of various types of polymers for different, unique and functional properties for medical sciences. This kind of nanoparticle has promising features for improving the quality of treatment, especially for modern nanomedicine. In recent years, researchers have looked at various factors such as particle size, morphology, material type, synthesis and optimization techniques to provide an optimal polymeric coating of nanoparticles for targeted drug delivery^29−31^. Polymer-coated nanoparticles are used in various therapeutic approaches such as drug release, tissue engineering and imaging. One of the first groups of polymer nanoparticles that can be used in the treatment of cancer is biodegradable polymers. Biodegradable polymer materials are known for their lower toxicity, the ability to create a drug release pattern and increase biocompatibility^32−35^.

In addition, lately layered double hydroxides (LDH) have attracted the attention of a large number of researchers. These layered structures are used as they are easy to prepare, inexpensive and versatile. The use of these nanolayer structures for human health is their use as drug delivery (drug carrier and delivery systems)^36,37^. Layered hydroxide compounds are a special type of coatings, whose crystalline structure is made of a two-dimensional (2D) unit by a weak force. An example of these materials is layered double hydroxides (LDHs) that have the anion exchange property^35,38^.

LDHs are substances that can be exploited for drug delivery. LDH 2D inorganic layers provide a relatively hydrophilic micro-environment for anticancer drugs. The beneficial effects of layered double hydroxides as the host for anticancer drugs can be done in several ways; The placement of medications in the 2D inorganic layers leads to a more protective effect, improving drug release in a more controlled fashion while improving low-solubility drugs and reducing side effects of the medications^39−41^.

**Figure 1:**
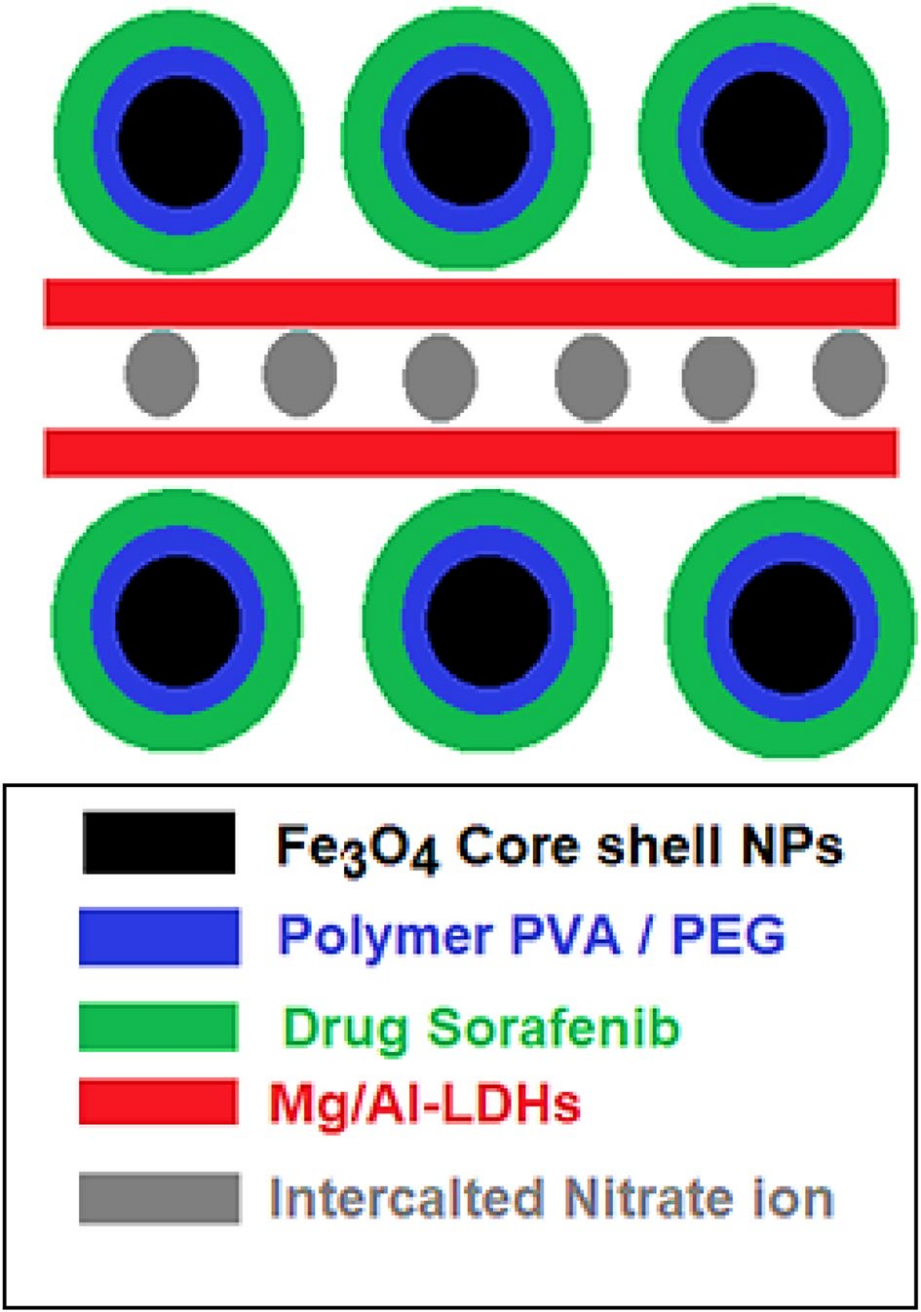
Schematic illustration of the designed core–shell nanocomposite.

In this paper, we discuss the synthesis and the physicochemical properties of magnetic nanoparticles coated with LDH and co-coated with polymers, PVA or PEG and loaded with sorafenib to be used for biomedical applications (Fig. 1). In addition, the effects of these 2 types of nanoparticles on normal fibroblast 3T3 and liver cancer HepG2 cell lines also will be described.

## Methods

### Materials and procedures

Ferric chloride hexahydrate (FeCl_3_·6H_2_O) and ferrous chloride tetrahydrate (FeCl_2_·4H_2_O) (99% purity) and ammonia solution (25%) were used as raw materials in this work. They were purchased from Merck (Darmstadt, Germany). In order to coat the surface of iron oxide nanoparticles with polymer, polyvinyl alcohol with 98% degree of hydrolysis, M.W. 60,000 and polyethylene glycol with average M.W. 6000 were acquired from Acros Organics (US). Aluminum nitrate (Al(NO_3_)_3_·9H_2_O) and magnesium nitrate (Mg(NO_3_)_2_·6H_2_O) with 99% purity supplied by ChemAR (Kielce, Poland). Sorafenib (C_21_H_16_ClF_3_N_4_O_3_) was sourced from Xi'an Yiyang Bio-Tech (China) at 98.5% purity. The solvent dimethylsulfoxide ((CH_3_)_2_OS) with high purity was obtained from Sigma Aldrich (St. Louis, MA, USA). Water (18.2 M Ω cm^−1^) was used in all the aqueous solutions.

### Experimental

The co-precipitation method was used to synthesize iron oxide nanoparticles. The process in which iron oxide nanoparticles are formed from the two soluble salts is shown according to the chemical Eq. (1), given below;

1 $$2{\mathrm{FeCl}}_{3} \cdot 6{\mathrm{H}}_{2} {\mathrm{O}} + {\mathrm{FeCl}}_{2} \cdot 4{\mathrm{H}}_{2} {\mathrm{O}}\,\mathop{\longrightarrow}\limits^{{}}\,{\mathrm{Fe}}_{3} {\mathrm{O}}_{4} + 8{\mathrm{NH}}_{4} {\mathrm{Cl}} + 20{\mathrm{H}}_{2} {\mathrm{O}}$$

At first, a 0.5 M aqueous solution of FeCl_3_·6H_2_O and FeCl_2_·4H_2_O were separately prepared at a ratio of 2:1 in a glass vessel and exposed to a continuous flow of nitrogen gas and was mixed with a mechanical stirrer. The temperature at 40 °C was set. After 10 min, 70 mL of ammonia was added to the system quickly. We continue to mix for 30 min. At this step, black sediments organized that indicated the formation of iron oxide particles. The sediments were isolated in three steps and washed with deionized water. Then, the washed precipitated with suitable concentration was dispersed in polymer suspension (containing 2 g of polyvinyl alcohol or polyethylene glycol in 100 mL ionized water) and placed in an autoclave for 24 h at 150 °C. After that, the black precipitate was collected by a centrifuge (5000 rpm for 5 min), washed 3 times to remove the remaining unbonded PVA or PEG during the coating process and then dried in an oven.

### Preparation of FPVASO-MLDH or FPEGSO-MLDH nanoparticles

The amount of 3 mL of coated iron oxide suspension (containing 5 g of iron oxide-PVA or PEG nanoparticles in deionized water) was added to a beaker containing 50 mL of the drug solution (3 g of sorafenib in dimethyl sulfoxide). Then the solution was stirred at room temperature for 24 h. In the next step, 6.5 g magnesium nitrate with 3.4 g aluminum nitrate was mixed with deionized water. While these materials were stirred with a magnetic stirrer, the sodium hydroxide was added dropwise to the solution to achieve pH 9–10. Eventually, 3 g of synthesized FPVASO/FPEGSO were mixed with a solution of Mg/Al-LDH at 40 °C and stirred to obtain a black precipitate. The final sample was washed with distilled water, centrifuged at 5000 rpm and dried in an oven.

### Instrumentation

X-ray diffraction was used to study the phase present in the sample, their crystal structure and the size of crystals. For this purpose, the XRD 6000 diffractometer machine from Shimadzu, Japan with CuKα radiation (λ = 1.5406 Å) at 40 kV and 30 mA in the range of 2°–80° were used. Samples used for this analysis were of powder form. Separation and measurement of sorafenib drug were performed by a high-performance liquid chromatography system (HPLC) using Alliance e2695, USA. The determination of morphology, shape, size distribution and estimation of particle size in micro and nano dimensions were analyzed by a transmission electron microscope (TEM, Hitachi H-7100, Tokyo, Japan). FESEM and EDAX images of the samples were recorded using a field emission scanning electron microscope, NOVA NANOSEM 230 model, California, USA. To determine the coating property of the particle surface by polymers, drug and LDH, the Fourier transform infrared spectroscopy (FTIR) was performed. To do this, a Thermo Nicolet 6700 (AEM, Madison WI, USA) with 0.09 cm^−1^ resolution with a wavenumber range of 500–4000 cm^−1^ was used. Samples of pure polymer, drug and LDH, and non-coated iron oxide nanoparticles were evaluated as reference. The magnetic properties of the nanoparticles were studied using the vibrating sample magnetometer (VSM) device, a Lakeshore 7404, Westerville, OH, USA. The thermogravimetric and differential thermogravimetric (TGA/DTG) analyses were obtained using a Mettler-Toledo of the Greifensee, Switzerland and were recorded in order to examine the thermal behavior of the nanoparticles. The amount of mass reduction was investigated in the effect of increasing the heat from ambient temperature to 1000 °C in the range of 20–1000 °C at a heating rate of 10 °C per minute. Inductively coupled plasma optical emission spectrometry (ICP-OES) for elemental quantization was done using a PerkinElmer spectrophotometer (Perkin Elmer, Wellesley, MA, USA). Model Optima 8300. The UV–Vis spectra in the solution condition were studied by a Lambda 35 ultraviolet–visible spectrophotometer (Perkin Elmer) apparatus to determine the drug release of the samples. The particle size distribution, as well as the mode of the particle size, was obtained by a dynamic light scattering (DLS, Malvern, NanoS, UK). The percentage of nitrogen, hydrogen and carbon of the magnetic nanoparticles was determined using CHNS elemental analyzer (LECO, TruSpec, Stockport, UK).

### MTT cell viability assay

The toxicity level of nanoparticles to the cell lines was assessed by cell viability test. Two types of cell lines including normal human fibroblast (3T3) and human hepatocellular carcinoma cells (HepG2) were used for this cytotoxicity evaluation. All the cells were grown by using Roswell Park Memorial Institute (RPMI) of 1640 medium (Nacalai Tesque, Kyoto, Japan) and the cell culture medium was supplemented with 10% fetal bovine serum (Sigma-Aldrich, MO, USA), 1% antibiotics containing 10,000 units/mL penicillin and 10,000 μg/ mL streptomycin (Nacalai Tesque, Kyoto, Japan). The cells were then incubated in humidified 5% carbon dioxide 95% room air at 37 °C. In the next step, the cell layers were harvested by using 0.25% trypsin/1 mM-EDTA (Nacalai Tesque, Kyoto, Japan) followed by seeded in 96-well tissue culture plates at 1.0 × 10^4^ cells/well for 24 h in an incubator to attach and 80% confluence achieved for treatment.

The cell viability and cytotoxicity was determined by 3-(4,5-dimethylthiazol-2-yl)-2,5- diphenyltetrazolium bromide (MTT)-based assay. For this purpose, the stock solutions were prepared by dissolving the compound in 1:1 of dimethyl sulfoxide (0.1%) and RPMI and the cells were treated by Cells were treated with pristine sorafenib (SO), FPEG-MLDH, FPVA-MLDH (nanocarriers), FPEGSO-MLDH, and FPVASO-MLDH (nanoparticles). The mixture was then diluted in the same medium to obtain various concentrations in the range of 1.25 to 100 μg/mL. Then, tested compounds were added until the final volume of 100 μL well upon the cell attachment to the respective wells after 24 h. After 72 h of incubation, 10 μL of MTT solution (5 mg/mL in PBS) was added in each well and incubated for 3 h prior to aspiration. After that, the purple formazan salt was dissolved by the addition of 100 μL of dimethyl sulfoxide to each well in the dark and room temperature. The intensity of the purple formazan solution reflecting the cell growth was subsequently measured by a microplate reader (Biotek LE800, Winooski, Vermont, USA) at a wavelength of 570 nm.

## Results and discussion

### X-ray diffraction

**Figure 2:**
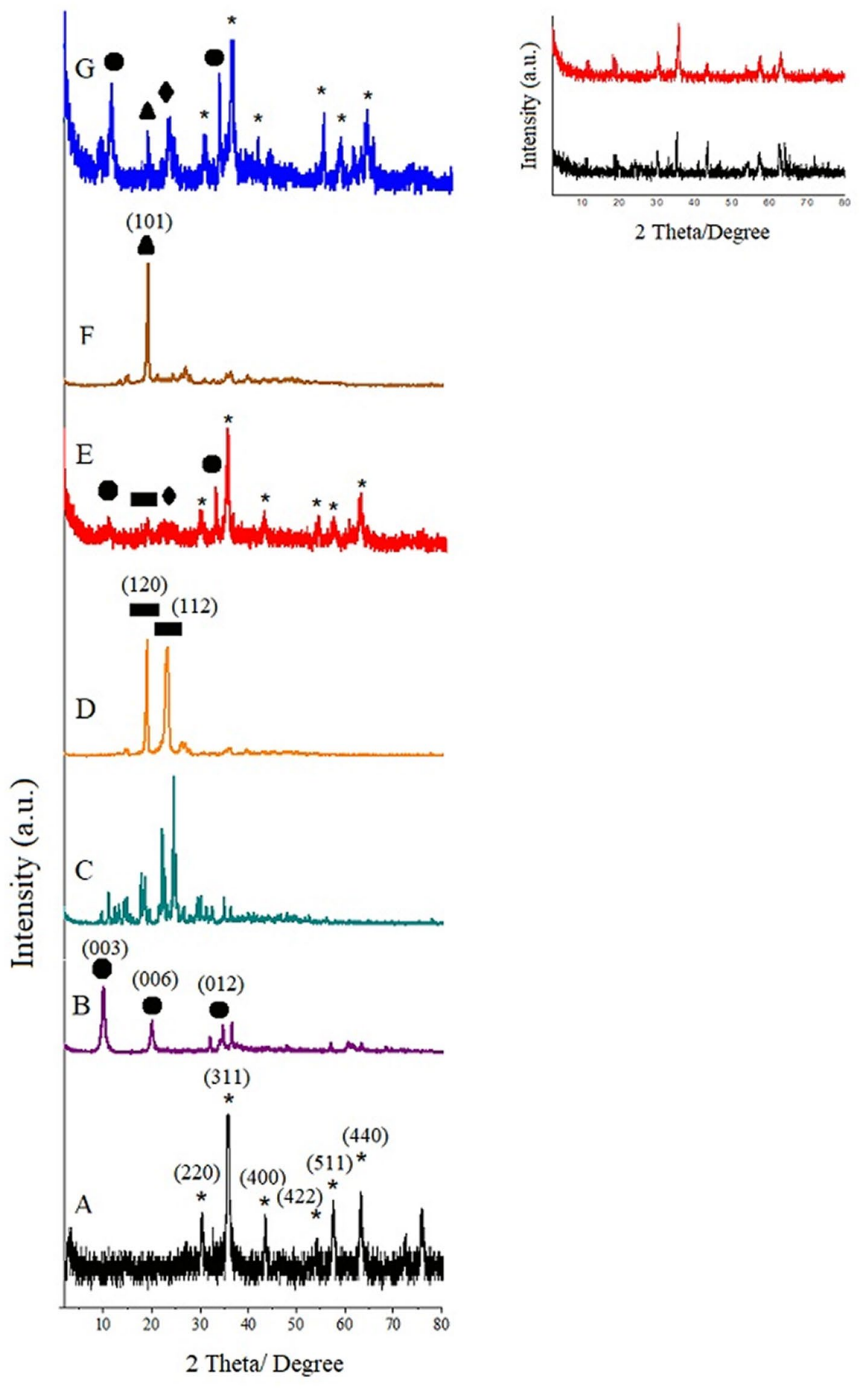
X-ray diffraction patterns for (**A**) iron oxide nanoparticles; (**B**) MLDH; (**C**) pure sorafenib; (**D**) pure PEG; (**E**) core–shell nanoparticles (FPEGSO-MLDH); (**F**) pure PVA; (**G**) core–shell nanoparticles (FPVASO-MLDH). Inset shows the XRD patterns of (**H**) FPEGSO and (**I**) FPVASO.

X-ray diffraction results of pure powder iron oxide nanoparticles, sorafenib, polyvinyl alcohol, polyethylene glycol, Mg/Al-LDH and the synthesized samples with different polymers and co-precipitation method are shown in Fig. 2. It is evident from the figure that in samples A, E and G only the iron oxide phase exist and no other subfamily is observed. Visible peaks with reasonably well-defined shape are related to reflections (220), (311), (400), (422), (511), and (440), which by matching all of these peaks with the JCPDS reference card number 85–1436 were confirmed as cubic and inverse spinal structure of the iron oxide nanoparticles^42^. The X-ray diffraction spectrum of samples d and f, at 2θ angles of 19.3°, 23.5° and 19.5° were interpreted as the main peaks of polyethylene glycol and polyvinyl alcohol, respectively. This can be attributed to the presence of these two polymers in the final samples. Furthermore, rather sharp peaks related to MLDH at the 2θ positions of 11.5°, 23.2° and 34.8° and sorafenib at the 2θ angles between 10° to 35° were present besides the iron oxide nanoparticles patterns in the final synthesized samples^43^. It should be noted that MLDH and sorafenib peaks overlap in the synthesized samples E and G because their 2θ positions are near to each other. Inset displays the XRD patterns of FPEGSO and FPVASO, suggesting that the basic coating agent (polymers) were of single-coated on iron oxide nanoparticles.

As shown in Fig. 2, X-ray diffraction patterns of the iron oxide nanoparticles core with the surface layer of polymer, drug and LDH do not have a significant change in the ratio of pure iron oxide nanoparticles, only reduced the intensity of the extent of the peak and increased the width of the peaks. That is because of the formation of the layer on the surface of iron oxide nanoparticles and it is due to their very small size. In addition, in Fig. 2E and G, additional peaks due to the polymer that is located on the surface of iron oxide nanoparticles are also visible. Basal spacings were increased from MLDH compared to the synthesized samples. This is related to the occupancy of the drug in the interlayer space of MLDH. Due to reflections for the magnetite and maghemite phase in the XRD patterns that are very close to each other, it cannot be ruled out that the synthesized samples are composed of a mixture of maghemite and magnetite phases^44,45^. However, because the magnetite synthesis was carried out under non-oxidizing conditions, the probability of the presence of the maghemite phase is low.

### Fourier transform infrared spectra

**Figure 3:**
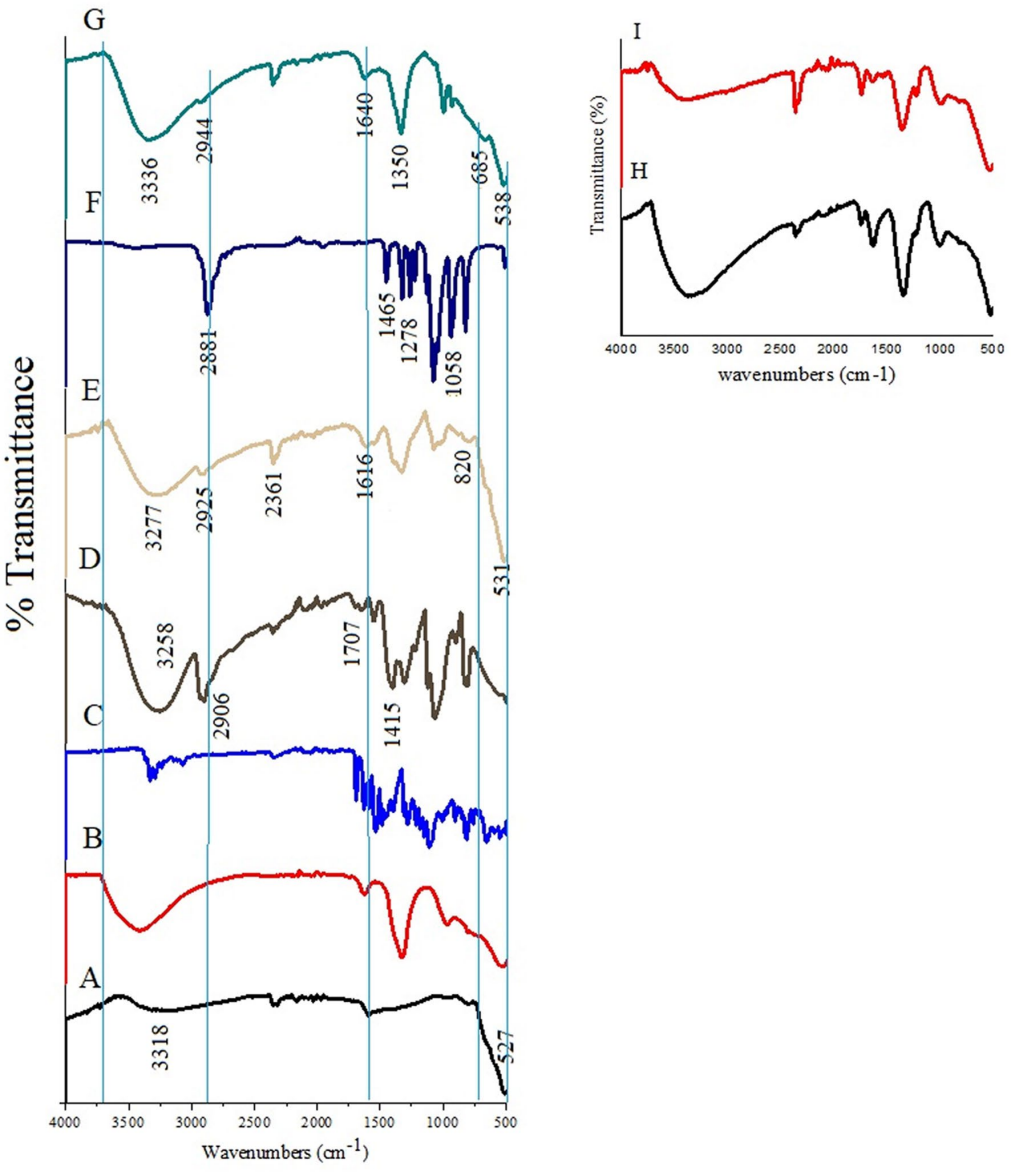
Fourier transform infrared spectra for (**A**) iron oxide nanoparticles; (**B**) MLDH; (**C**) pure sorafenib; (**D**) pure PVA; (**E**) core–shell nanoparticles (FPVASO-MLDH); (**F**) pure PEG; (**G**) core–shell nanoparticles (FPEGSO-MLDH). Inset shows the FTIR spectra of (**H**) FPEGSO and (**I**) FPVASO.

In order to confirm the formation of the polymer and drug (Fig. 3C) layers on the surface of the iron oxide core, as well as the formation of LDH (Fig. 3B) as shell and its structural identification, Fourier transforms infrared spectroscopy was carried out. The FTIR spectra are shown in Fig. 3. This figure shows the absorption of the iron oxide nanoparticles, which were synthesized by the co-precipitation method, in the range of 4000–500 cm^−1^ (Fig. 3A). The intense absorbance bands at around 410–480 cm^−1^ and also 581–629 cm^−1^ are referred to as the iron oxide superparamagnetic nanoparticles core due to stretching Fe–O group. The bands are expected to be observed at 370 cm^−1^ and 570 cm^−1^, respectively, but due to their small size (at nano-sized regime), they were shifted to lower wavenumbers^46^.

The study of the PEG FTIR spectrum (Fig. 3F) demonstrated that bands observed at 1058, 1278 and 1465 cm^−1^ represent an ether, C-O–H stretching and C-H bending vibrations, respectively. A sharp peak at 2881 cm^−1^ illustrated the C-H stretching vibrations which are available in PEG structure. The results of FTIR spectrum for PVA (Fig. 3D) indicate four sharp broad bands between 3550–3100 cm^−1^, 2840–3000 cm^−1^, 1700–1750 cm^−1^ and 1400–1461 cm^−1^ wavenumbers, which are associated to the O–H stretching from the intermolecular and intramolecular hydrogen bonds, the C–H stretching due to the alkyl groups and the C=O and C–O stretching due to the acetate groups and C–H_2_ bonds, respectively^42^. As can be seen from the FTIR spectra (inset of the figure) there are sharp absorption peaks that are related to polymer, sorafenib and iron oxide groups that are present in FPEGSO (Fig. 3H) and FPVASO nanoparticles (Fig. 3I).

The FTIR spectra of samples E and G, along with loading of anti-cancer drug sorafenib, (Fig. 3E,G) show bands at 820 cm^−1^ and 686 cm^−1^ which are overlapped with a band related to –C–F bonding. Also, vibrations at 1616 cm^−1^ and 1640 cm^−1^ are due to the C=C in the alkene. Sharp bands at 3277 cm^−1^ and 3336 cm^−1^ are due to the N–H bond of the amine stretching, bands of medium intensity at 2925 cm^−1^ and 2944 cm^−1^ are representative of the presence of C–N bond in aminopropyl groups, respectively. The presence of all these bands indicates the success of the polymer/LDH/sorafenib coating which was loaded onto the surface of the iron oxide nanoparticles. Furthermore, bands at 531 cm^−1^ and 538 cm^−1^ confirmed the formation of the iron oxide core in sample E and G, respectively.

Bands at 2361 cm^−1^ and 1350 cm^−1^ are responsible for the C–H bending and the C–H stretching vibration in both the synthesized samples, respectively. This indicated the formation of the bond between shell (polymers) and the core. The absorption bands at 1046 cm^−1^ and 947 cm^−1^ belong to the O–M–O lattice vibration (SO was intercalated into MLDH by co-precipitation and ion exchange method). It should be considered that bands' intensity has been reduced and shifted owing to interactions between LDHs and the synthesized nanoparticles^47,48^.

### Magnetic properties

**Figure 4:**
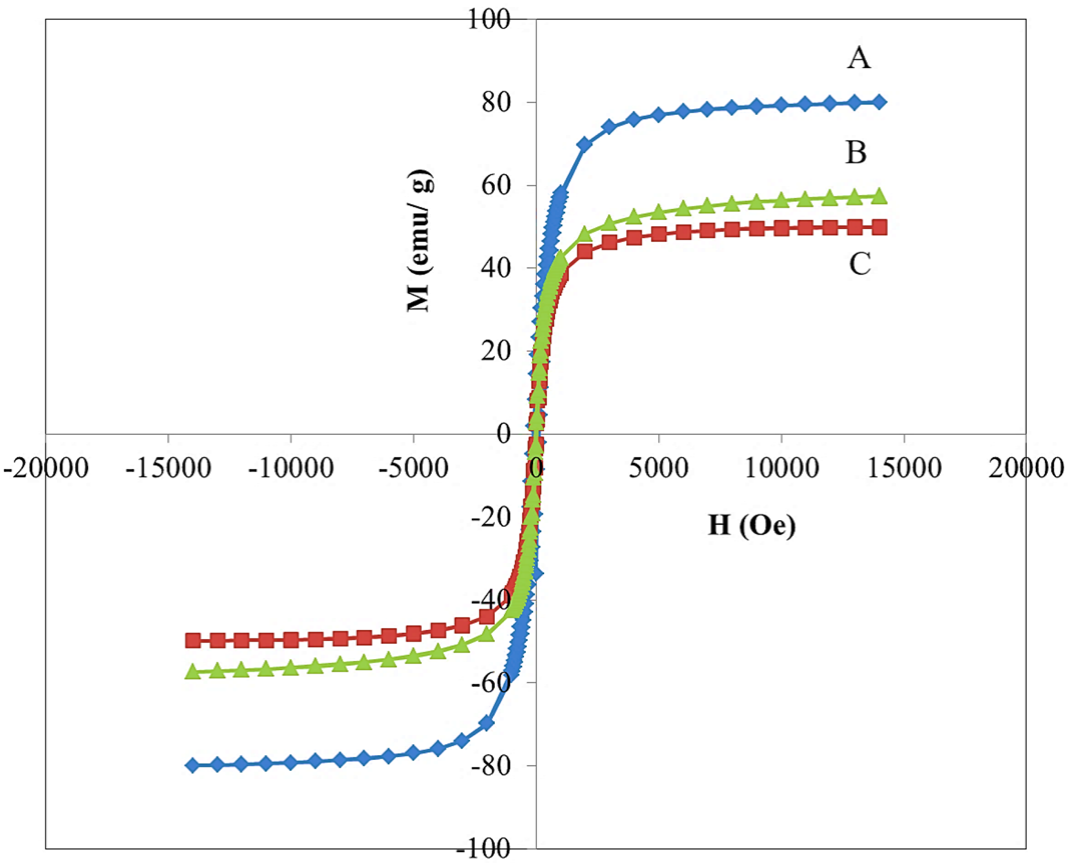
Magnetization curves of (**A**) iron oxide nanoparticles; (**B**) core–shell nanoparticles (FPEGSO-MLDH); (**C**) core–shell nanoparticles (FPVASO-MLDH). Notes: The data is presented in terms of Ms, mass magnetization (emu/g) versus H, applied magnetic field (Oe).

The results of the study using vibrating sample magnetometer (VSM) analyses are displayed in Fig. 4 and the values for saturation magnetization (Ms), remnant magnetization (Mr) and high coercivity (Hci) are presented in Table 1. The results confirm the superparamagnetism behavior of the samples due to the presence of the core, iron oxide nanoparticles. Based on the value of saturation magnetization, it is clear that the nanoparticles have magnetic properties. At room temperature, the hysteresis loops were not observed. The magnetic saturation values of FNPs, FPEGSO-MLDH and FPVASO-MLDH were found to be 80, 57 and 49 emu/g, respectively.

**Table 1:** The magnetic property of FNPs, FPEGSO-MLDH and FPVASO-MLDH.

Samples	Ms (emu/g)	Mr (emu/g)	Hci (G)
FNPs	80	1.45	11.53
FPEGSO-MLDH	57	2.74	19.33
FPVASO-MLDH	49	2.49	20.93

The saturation magnetization of samples B and C compared to pure iron oxide nanoparticles with a value of 80 emu/g showed they have lower saturation magnetization. Three main reasons for this are due to the presence of non-magnetic layers at the surfaces of the core, distribution and dispersion of cations, as well as spin effects. Figure 4 shows the hysteresis curve of the samples at room temperature and in the fields of 20,000 to − 20,000 Oersted. As it was observed, the magnetization curve of the particles passes from the source, and in them, there are no coercivity field strength, hysteresis and remanent magnetization (Mr). Therefore, it is noteworthy that all the synthesized nanoparticles are superparamagnetic at room temperature^5,6^. As expected, the formation of polymer-drug-LDH shells on iron oxide nanoparticles led to a weakening of the magnetic property, but the magnetic saturation obtained is still acceptable for use in medical applications. In both the synthesized samples, the remanence and coercivity were apperceived, which acknowledged the superparamagnetic property. This means that after the removal of the magnetic field, the nanoparticles did not maintain any magnetism property^14,49^.

### Thermogravimetric analyses

**Figure 5:**
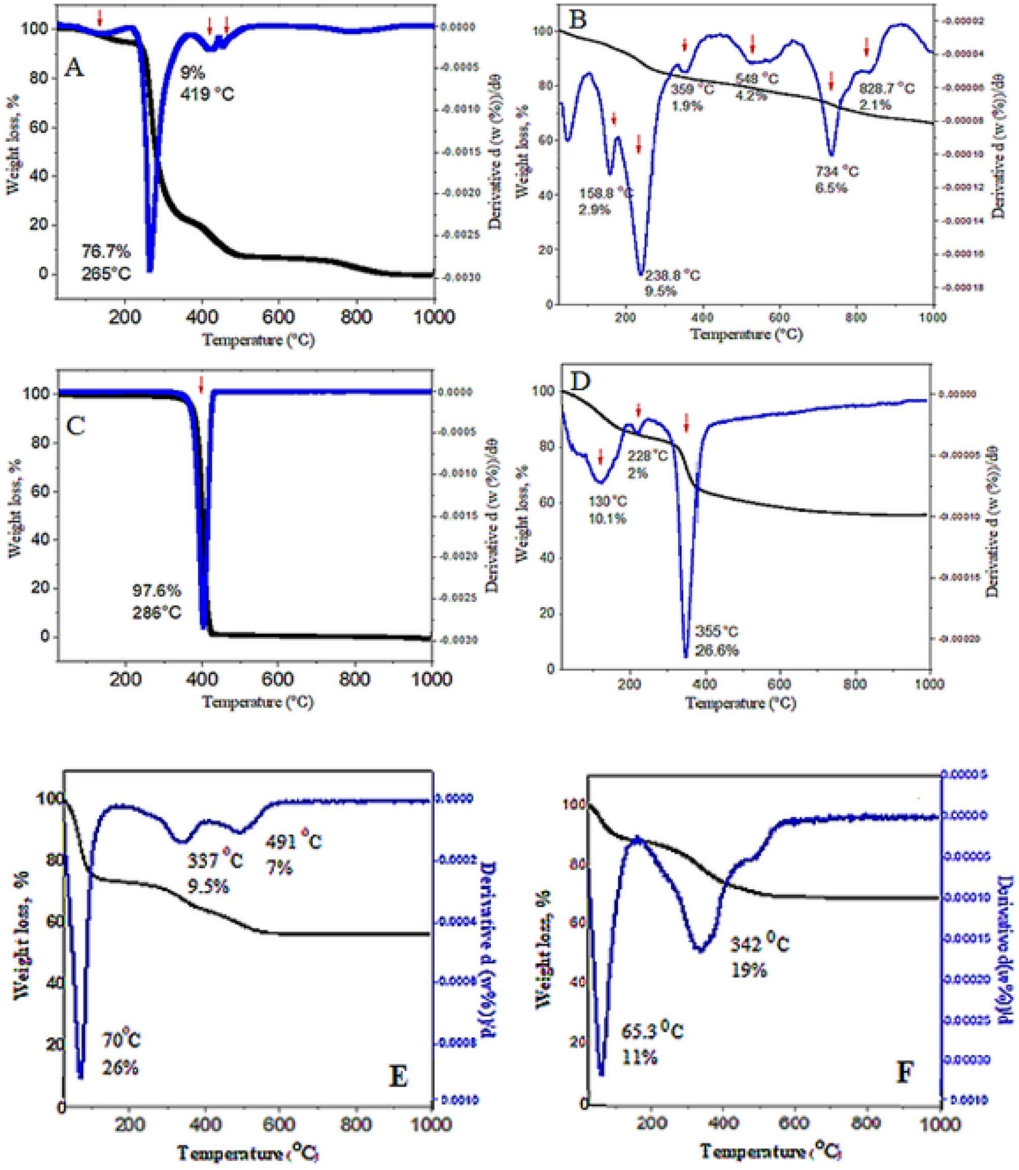
Thermogravimetry analyses of (**A**) pure PVA; (**B**) core–shell nanoparticles (FPVASO-MLDH); (**C**) pure PEG; (**D**) core–shell nanoparticles (FPEGSO-MLDH); (**E**) FPEGSO; (**F**) FPVASO. Note: The red arrows point to the weight loss.

In order to study the thermal behavior of the samples coated with polyethylene glycol and polyvinyl alcohol, pure polyvinyl alcohol (Fig. 5A) and polyethylene glycol (Fig. 5C) the weight loss curve upon temperature increased (TGA and DTA curves) are shown in Fig. 5. In this way, the amount of mass reduction owing to the unstable polymeric coating due to the heating process can be obtained.

Based on Fig. 5B, there are weight losses from a temperature of about 40 °C to 300 °C which can due to the degradation of polyvinyl alcohol chains. In other words, polyvinyl alcohol starts to burn out and all its chains were destroyed at about 500 °C. The next region of temperature decrease is from 650 °C to about 900 °C, which was due to two factors:

1. Conversion of iron oxide nanoparticles to Wüstite (FeO) under the absence of oxygen, but in the presence of residual carbon from the polyvinyl alcohol chains^50,51^. This conversion occurs according to reaction 2 below.
  2 $${\mathrm{C}} + {\mathrm{Fe}}_{3} {\mathrm{O}}_{4} \,\mathop{\longrightarrow}\limits^{{}}\,3{\mathrm{FeO}} + {\mathrm{CO}}$$
2. Adhesion or agglomeration of the particles, where the remaining hydroxide group in the structure of the nanoparticles that evaporate at this temperature range, resulting in weight loss.

Figure 5D shows the molar mass of the adsorbed polyethylene glycol on the surface of iron oxide nanoparticles with about 4% weight loss as observed in the TGA curve which may be due to the evaporation of the adsorbed H_2_O. A sharp weight loss with 26.6% is due to the PEG decomposition as indicated in the TGA profile at 355 °C^42^.

About 2.9% and 10.1% of the initial weight for samples B and D, respectively were lost at around 200 °C. The onset of weight loss in sample B was 158.8 °C, whereas in sample D the temperature was reduced to 130 °C. The cause of these weight loss is mainly due to the evaporation of the tough outer layer or the conversion of organic elements due to carbonization. The increase in molecular weight and the length of the carbon chain are associated with weight loss. Furthermore, a major decomposition at 238 °C and 228 °C (with a weight loss of 9.5% and 2%, respectively) are ascribed to the decomposition of the hydroxide layer hydroxylation. The X-ray diffraction pattern of the synthesized samples coated with PEG and PVA did not demonstrate the presence impurity, as no new reflections were observed.

Also, the thermal behavior of FPEGSO and FPVASO was measured (Fig. 5E,F). As can be seen in Fig. 5E,F, no weight loss was recorded due to the dehydroxylation of LDHs. The final core–shell nanoparticles show improved thermal stability than the basic nanoparticles due to the hydrogen bonding between the sorafenib and the host, LDHs nanolayers. For instance, the de-hydroxylation decomposition of the FPEGSO occurred at a lower temperature (372 °C) than the one in the FPEGSO-MLDH (466 °C). Figure 5B shows polyvinyl alcohol (12.4% of the whole), iron oxide (8.6% of the whole), sorafenib (21% of the whole) and MLDH (12.5% of the whole) while Fig. 5D shows polyethylene glycol (26.6% of the whole), sorafenib (17.9% of the whole) and MLDH (10.1% of the whole) depending on their different decomposition temperature or evaporation.

In the TGA curve, the weight loss of the sample is primarily due to the degradation and decomposition of the shells. Qualitative analysis shows that both polymers molecules were adsorbed on iron oxide nanoparticles surfaces. By considering the stability of iron oxide nanoparticles up to 700 °C, the amount of bond stability between the core and the shell can be evaluated. As can be seen, the weight loss process was uniform, indicating the shell did not instantaneously separate from the mineral core on the heating process, suggesting an ideal bonding between the core and the shell. The formation of the hydrogen bond between polymers and iron oxide nanoparticles prevent the other bonds to be further form to the Fe_3_O_4_ nanoparticles.

### Surface properties

**Figure 6:**
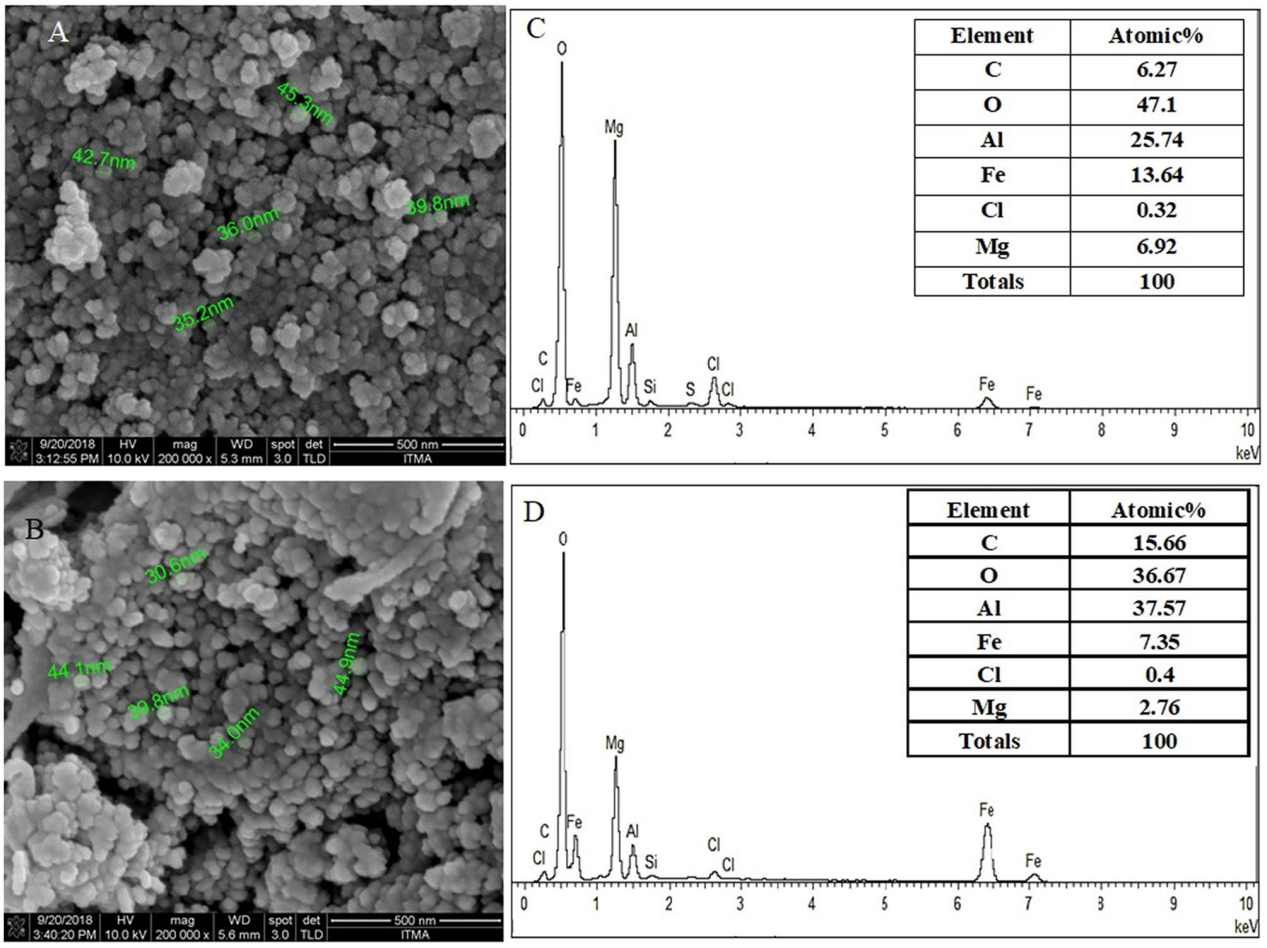
FESEM of (**A**) core–shell nanoparticles (FPEGSO-MLDH); (**B**) SEM–EDX spectra; (**C**) core–shell nanoparticles (FPVASO-MLDH); (**D**) SEM–EDX spectra. *The sampel holde ris made up of aluminum and this leads to a high percentage of aluminum as depicted in the spectra. Thus, it is not a reliable indication of aluminium content in the sample.

**Figure 7:**
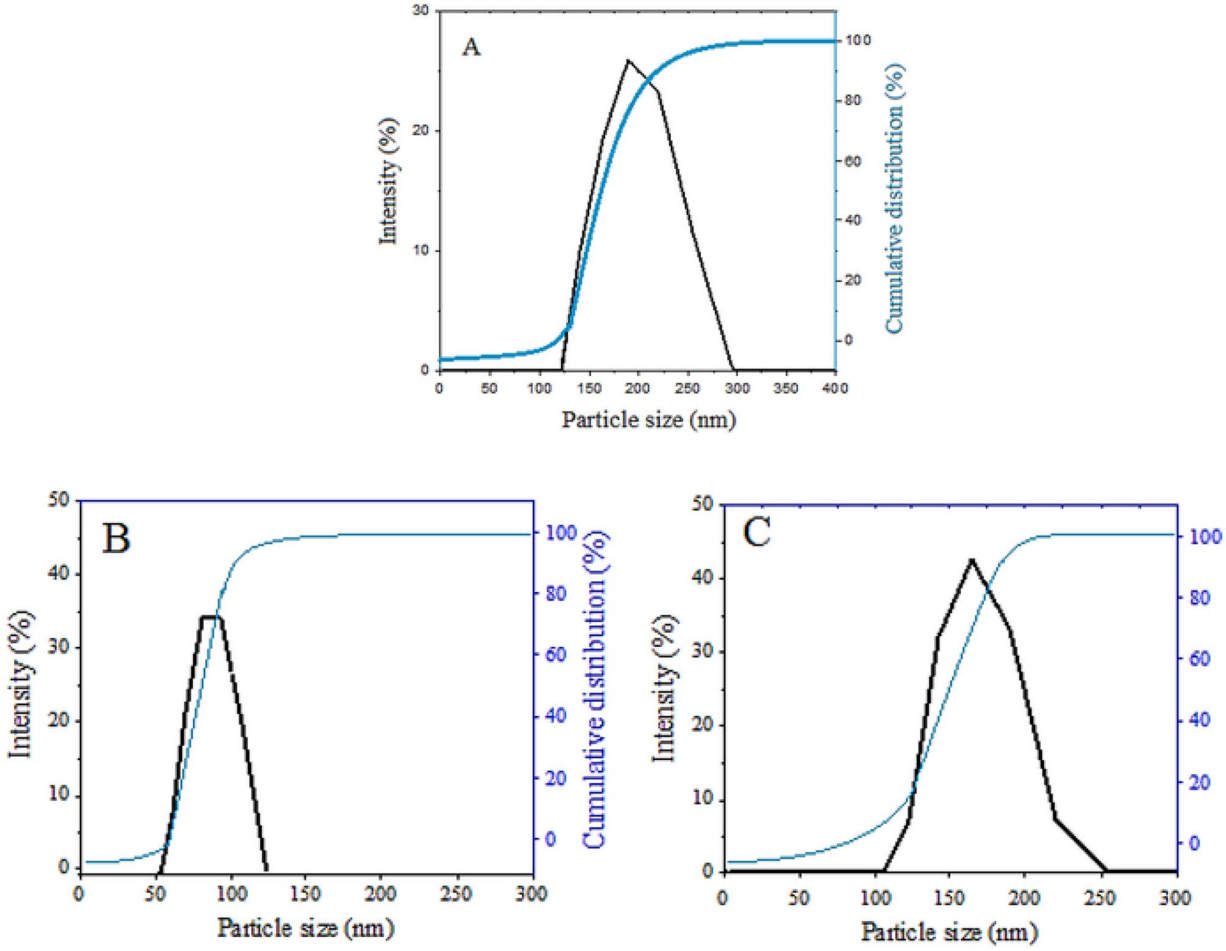
Cumulative and particle size distribution of (**A**) the iron oxide nanoparticles; (B) core–shell nanoparticles (FPEGSO-MLDH) and (C) core–shell nanoparticles (FPVASO-MLDH).

Shape and surface morphology of coated magnetic nanoparticles were determined using a field emission scanning electron microscope (FESEM) with 200.000 × magnifications (Fig. 6). In addition, the results of the dynamic light scattering (DLS) test on the samples can be viewed in Fig. 7. The FESEM analysis of the samples indicates the effect of the coatings agents on the morphology of the iron oxide nanoparticles and showed that the nanoparticles were spherical and had a narrow particle size distribution. Nanoscale size and particle size distribution can be contributed to the stability of iron oxide nanoparticles suspension, for their use in drug delivery applications.

Iron oxide nanoparticles were highly absorbable during the germination because of their extremely energetic and active surface. In the presence of a surface agent, the surface is available for embranchments attack on the surface and the iron oxide nanoparticles are rapidly surrounded by the surface agent. Therefore, the growth of particles was constrained, particle size remained small and the agglomeration was prevented. This demonstrated that the presence of a surface coating could reduce the particle size. In the present method, polymers had immediately peched onto the surface after the formation of iron oxide nanoparticles and indirectly they prevented particle agglomeration.

It is worth noted that the presence of agglomerates is unavoidable because of interparticle interactions as well as the presence of the magnetic field during washing, forms agglomerates and secondary structures. With the addition of surface coatings, these structures are partially broken and the polymer is placed on their surface. It is worth noting that the replacement of interlayer-bound drugs with nitrate anions during the exchange process could cause clump and agglomeration of particles through attachment to the surface.

Figure 6 demonstrates the SEM–EDX analysis and data are summarized in the table. As can be seen in the table, iron, oxygen, carbon, aluminum and magnesium elements are present, which confirmed the presence of all compounds in the synthesized nanoparticles. Based on the table, the atomic percentage of iron oxide, oxygen, carbon, aluminum and magnesium are 13.6%, 47.1%, 6.2%, 25.7% and 6.9% for the iron oxide nanoparticles coated with PEG compared to 7.3%, 36.6%, 15.6%, 37.5% and 2.7%, respectively for the iron oxide nanoparticles coated with PVA (Fig. 6D). It should be noted that the observation of carbon peak is due to the presence of polymers, aluminum and magnesium that have been arisen from the presence of the Mg/Al-LDH phase and the presence of iron peak was caused by the iron oxide core.

The size distribution and the average particle size of the solid nanoparticles dissolved in methanol and their hydrodynamic size were obtained by the dynamic light scattering (DLS) analysis (Fig. 7). As shown in Fig. 7A, monomodal particle size distribution of the raw iron oxide nanoparticle shows a peak at 198 nm. Moreover, the effect of the presence of polymer (PEG and PVA), LDHs and drug on the average size distribution of iron oxide nanoparticles also was investigated. The cumulative distribution frequency related to the synthesized sample with PEG displays a narrow size distribution between 58–105 nm with a monomodal particle size distribution with a peak at 81 nm (Fig. 7B). Thereupon, the result for sample 6C where PVA was used as the coating agent, also indicates a narrow size distribution in the range of 122–220 nm with ta monomodal particle size distribution with a peak at 171 nm. Henceforward, these sizes confirmed the nanoscale of the synthesized particles.

As shown in the figures, the hydrodynamic size of the synthesized nanoparticles coated with polyethylene glycol was smaller than the other samples, and the particle size distribution was also narrower. PEG is known as a non-ionic polymer with a regular chain structure that readily absorbs onto the surface and can significantly reduce the rate of particle growth. This organic additive acts as a coating agent and reduces the rate of nucleation.

### High-resolution transmission electron microscopy

**Figure 8:**
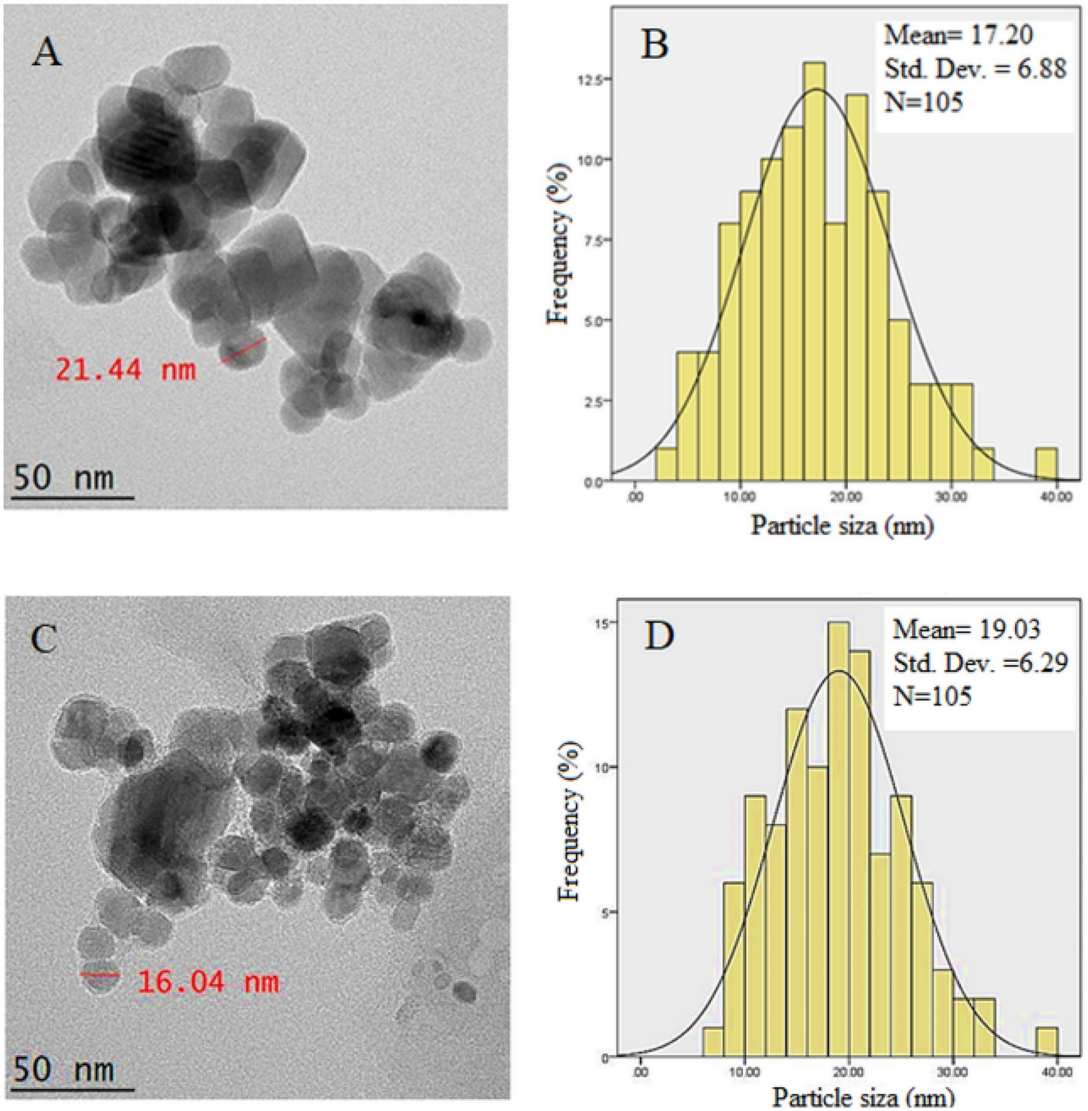
Transmission electron micrographs for (**A**) core–shell nanoparticle (FPEGSO-MLDH) (50 nm bar); (**C**) core–shell nanoparticle (FPVASO-MLDH) (50 nm bar); (**B**,**D**) their particle size distribution.

High-resolution transmission electron microscopy (HRTEM) was used to evaluate the actual size of the nanoparticle, to study the inner structure such as crystal structure and morphology of the synthesized nanoparticles and to look at the coating condition. This is shown in Fig. 8. As shown in the figure, the nanoparticles were generally of spherical shapes (Fig. 8A,B). The darker particles visible in figures are due to iron oxide nanoparticles and the bright halos around them are polymer, drug and carrier coatings. It can also be concluded that the results of TEM showed the formation of continuous and uniform structures of the nanoparticles with almost similar size. As can be seen from the figures, the nanoparticles are of single-crystal and since the iron oxide particles are smaller than 50 nm is monopolar, so the synthesized nanoparticles were also monopolar.

Using image analysis software from 100 particles chosen randomly, the particle size was obtained. The mean diameter of the synthesized nanoparticles coated with polyethylene glycol was found to be around 17 nm with narrow size distribution compared to 19 nm for the nanoparticles coated with polyvinyl alcohol. This indicates the type of polymer coating, to some extent could affect the size of the resulting nanoparticles. In addition, the PVA-coated nanoparticles show they are more aggregated and clustered together. On the other hand, the PEG-coated nanoparticles are relatively more separated from each other and gave a more uniform size compared to the PVA-coated nanoparticles.

### Percentage loading of sorafenib

The isolation, detection and quantification of constituents in the substrate such as a drug is performed by a chemical analysis called High-performance liquid chromatography (HPLC). In order to measure the percentage of drug loading, the samples were first diffused in methanol exposed to ultrasonic irradiation for 1 h (40% powder in 40 °C) and standards of sorafenib were prepared at various concentration of 0, 25, 50, 100, 150 and 200 ppm. Finally, the calibration curve is plotted with an R^2^ of 0.99.

**Figure 9:**
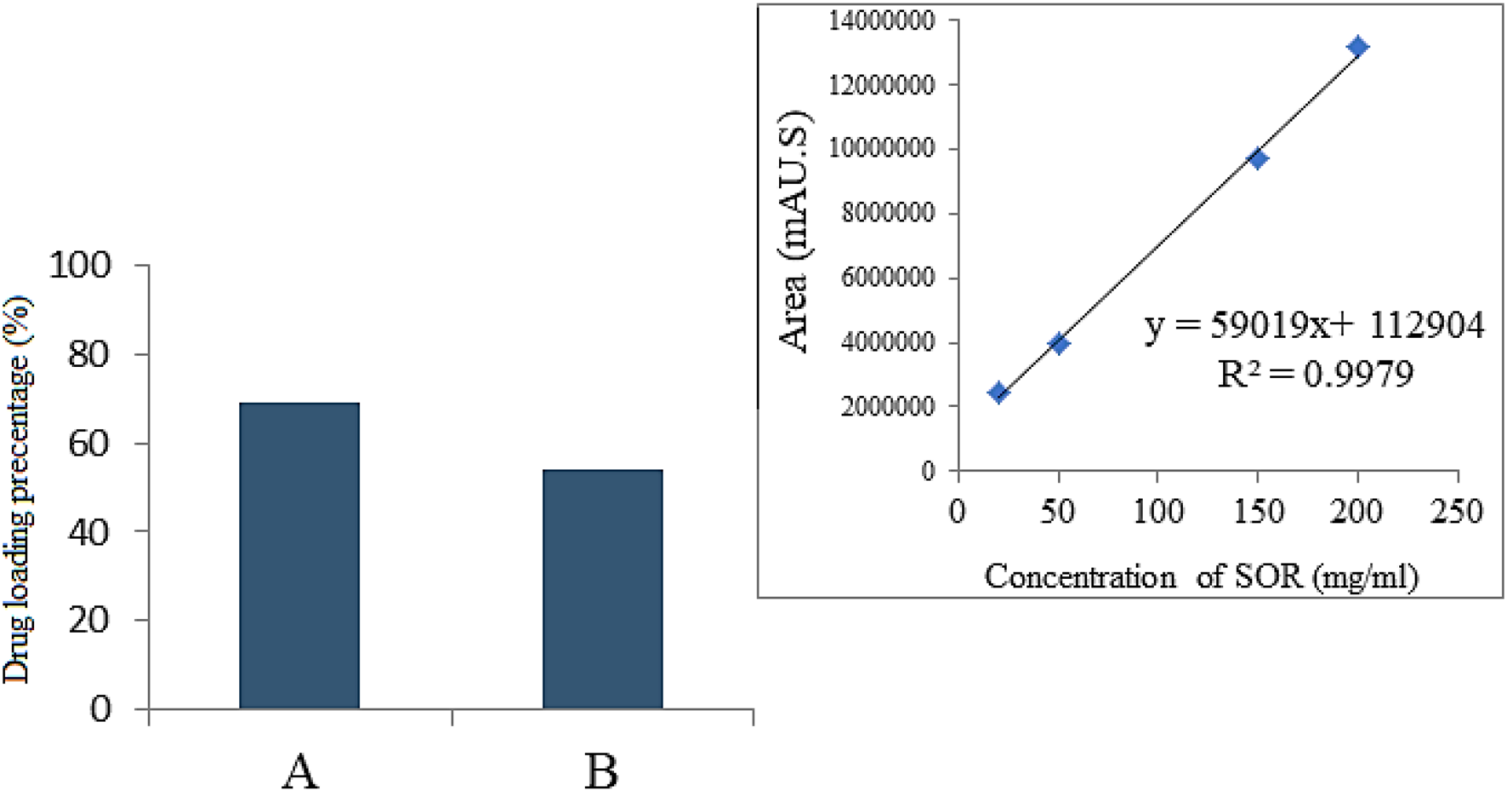
HPLC plot of (**A**) the nanoparticles co-coated with PEG; (**B**) the nanoparticles co-coated with PVA.

Previously reported method was adopted for the quantification of Sorafenib with a limit of detection of 97 ng/mL. To measure the percentage of drug-loaded from a mobile phase with a relative ratio of 55/45 (v/v) of phosphate buffer at pH 7.4: CH_3_CN with the flow rate of 1.5 mL.min^−1^ was used in this work. The HPLC has Column C_18_ Agilent in a dimension of 3.9 × 150 mm, the particle diameter of 4 μm, at the column oven temperature of 30 °C and an injection volume of 2 µl and photodiode detector array (PDA), Waters HPLC 2695 separation module components. As can be seen in Fig. 9, the chromatographic manner was shown a clear pattern in the rate of change in drug loading percentage, which may indicate chromatographic accuracy and efficiency. It was found that the drug loading in the sample coated with polyvinyl alcohol is lower than the one coated with PEG, with a value of 54% for the former compared 69% for the letter.

### Elemental analyses

CHNS elemental analyzer is a method for determining the percentage of carbon, hydrogen, nitrogen and sulfur in a sample. For this purpose, the gases from the combustion of the sample are examined by a gas chromatography technique. The calculated values are shown in Table 2. In this analysis, the presence of carbon and hydrogen indicates the successful coating of polymers onto magnetite nanoparticles. The presence of nitrogen is due to its presence in the molecular structure of the drug, sorafenib.

**Table 2:** Elemental composition of the nanoparticles obtained from CHNS and ICP-OES analyzers.

Sample/%	*C	*H	*N	Mg	Al	Fe
FNPs	0.02	0.54	1.02	–	–	47
MLDH	–	3.18	4.64	7.3	4.5	–
SOR	52.9	5.01	19.84	–	–	–
PEG	52.69	8.98	1.64	–	–	–
FPEGSO-MLDH	3.66	3.96	0.01	2.7	2.9	19.8
PVA	52.05	8.68	1	–	–	–
FPVASO-MLDH	2.18	1.93	0.06	3.7	4.2	25.7

ICP-OES was used for the elemental analysis and to measure the elements with a detection limit at the ppm-level. Based on Table 3, the percentage of magnesium, aluminum and iron in sample FPEGSO-MLDH was found to be 7%, 4% and 45%, respectively. It was found the Mg, Al and Fe contents are 5%, 3% and 4%, respectively for sample FPVASO-MLDH. These results indicate that magnesium, aluminum and iron were present in both samples, MLDH and ION at detectable levels. The presence of Fe confirmed the existence of ION as the core.

**Table 3:** Percentage of elements in the nanoparticles.

Sample/%	*C	*H	*N	Mg	Al	Fe
FNPs	–	0.54	0.07	–	–	0.8
MLDH	–	3.1	0.3	0.3	0.17	–
SOR	4.4	5.0	1.4	–	–	–
PEG	4.4	8.9	0.1	–	–	–
FPEGSO-MLDH	0.3	3.9	–	0.1	0.1	0.36
PVA	4.3	8.6	0.07	–	–	–
FPVASO-MLDH	0.1	1.9	–	0.1	0.16	0.46

### In vitro release of Sorafenib from the magnetic nanoparticles

**Figure 10:**
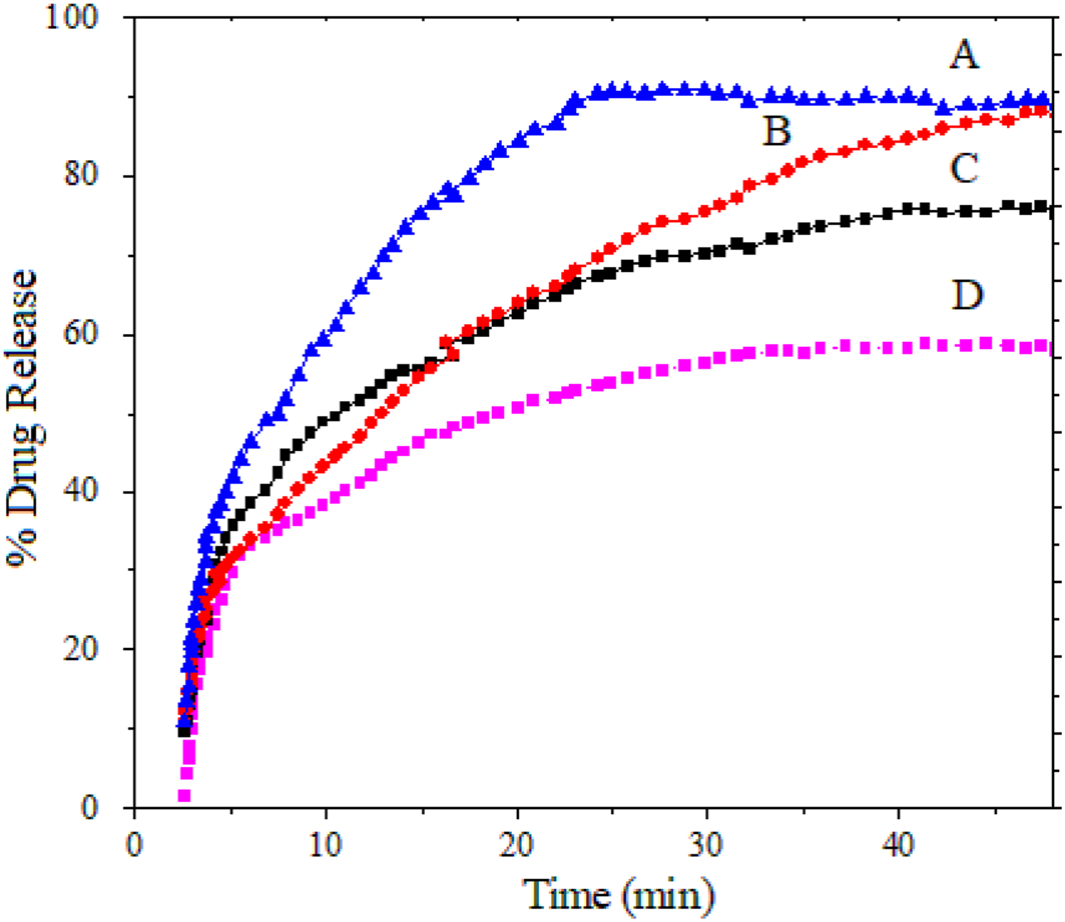
Release profiles of sorafenib from its physical mixtures (**A**) FPEGSO-MLDH; (**B**) FPVASO-MLDH in phosphate-buffered solution at pH 4.8; (**C**) FPEGSO-MLDH; (**D**) FPVASO-MLDH in phosphate-buffered solution at pH 7.4.

**Figure 11:**
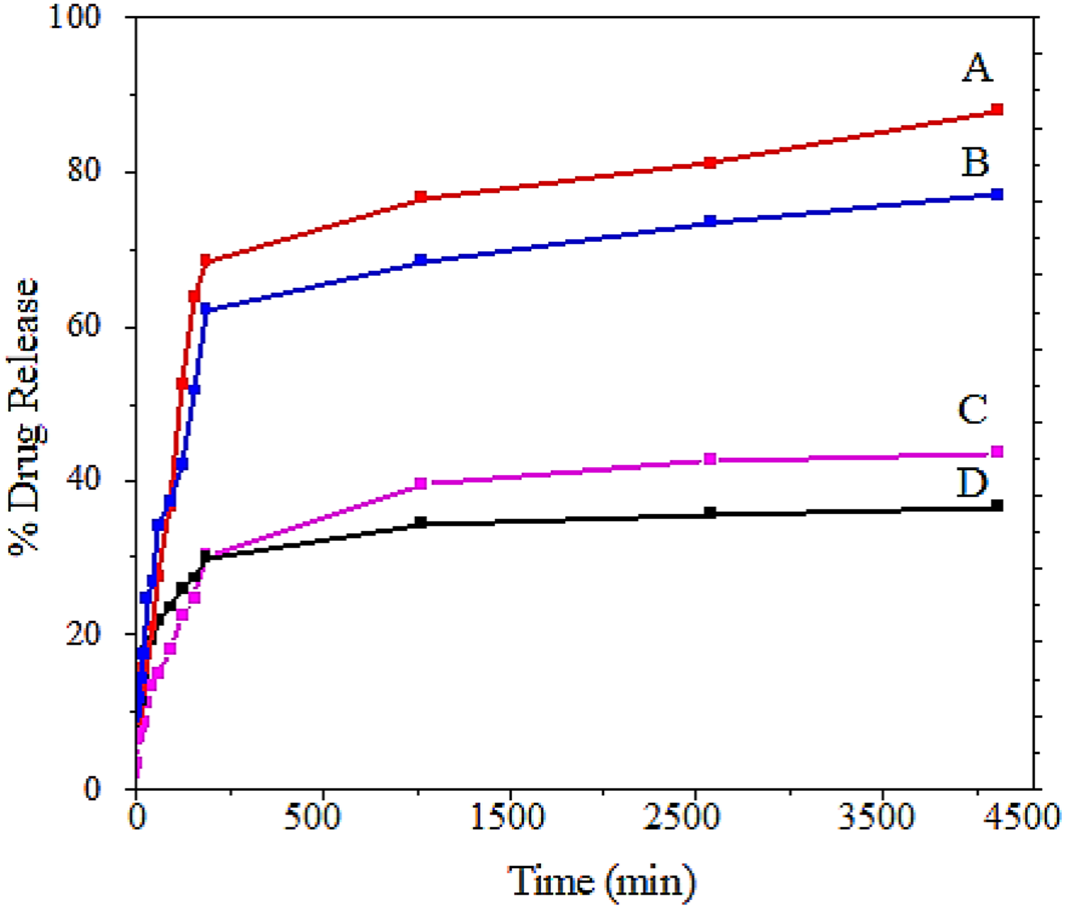
Sorafenib release profiles from (**A**) FPEGSO-MLDH; (**B**) FPVASO-MLDH in phosphate-buffered solution at pH 4.8; (**C**) FPEGSO-MLDH; (**D**) FPVASO-MLDH in phosphate-buffered solution at pH 7.4.

To investigate the release profiles of the physical mixture of sorafenib and magnetic nanoparticles in phosphate-buffered saline (PBS), solutions were prepared at 2 pHs, namely 7.4 and 4.8, to mimic normal and tumor environments and the ultraviolet–visible spectrometer instrument was used and the results are given in Fig. 10. In the traditional methods of drug intake, drug levels increase after intake and then decrease until the next intake. In contrast, in the controlled drug delivery method, drug levels remain constant for a long period in the bloodstream. However, one of the disadvantages of this method is the sudden or burst release of the drug at the early stage of drug intake. In the physical mixture, where sorafenib and magnetic nanoparticles coated with polyvinyl alcohol or polyethylene glycol were mixed, the initial drug release was rapid, followed by a slow one and thereafter a steady release was observed. This trend is visible in Fig. 11. During the first 20 min, around 83% and 82% of the drug was completely released from the magnetic nanoparticles coated with PEG or PVA into PBS solution at pH 4.8, respectively. Conversely, for samples, C and D, 69% and 58% of sorafenib were released into the phosphate-buffered saline solution at pH 7.4, respectively.

Polyvinyl alcohol and polyethylene glycol are both hydrophilic, but the drug, sorafenib is hydrophobic. Due to their incompatibility, the migration of drug molecules to the surface of the nanoparticles is increased, which then easily diffuses into the aqueous phase, leading to the rapid initial release. As polyethylene glycol is more soluble in water, it may cause more drugs to be migrated outward, and therefore the release of the drug is faster. Obviously, as the amount of drug loaded onto the nanoparticles was increased, the amount of drug released will be also increased. In addition, due to the dissolution of the nanoparticles’ shell in solution and the breaking of the bonds between the polymer and the drug, the release of sorafenib was expectedly faster in the solution at pH 4.8^52^.

### Kinetics release of sorafenib from the nanoparticles

The release kinetics of sorafenib from the magnetic nanoparticles into PBS at pH 7.4 and 4.8 were measured using several kinetic models by ultraviolet–visible spectroscopy. There are several models for describing release kinetics; the first-order, the pseudo-second-order and the parabolic diffusion.

3 $${\mathrm{In}}\left( {{\mathrm{q}}_{{\mathrm{e}}} - {\mathrm{q}}_{{\mathrm{t}}} } \right) = {\mathrm{In}}\;{\mathrm{q}}_{{\mathrm{e}}} - {\mathrm{kt}}$$

4 $${\mathrm{t/q}}_{{\mathrm{t}}} = 1/{\mathrm{k}}_{2} {\mathrm{q}}_{{\mathrm{e}}}^{2} + {\mathrm{t/q}}_{{\mathrm{e}}}$$

5 $$\left( {1{-}{\mathrm{M}}_{{\mathrm{t}}} /{\mathrm{M}}_{0} } \right)/{\mathrm{t}} = {\mathrm{k}}_{{\mathrm{t}}}^{ - 0.5} + {\mathrm{b}}$$

where q_e_ is the equilibrium release rate, q_t_ is the release rate at time t, k is a constant corresponding to release rate, M_o_ is the drug content remaining at release time 0 and M_t_ is the drug content remaining at release time t.

Based on the fitting, among the three mentioned models, the pseudo-second-order model has a high correlation coefficient with the experimental data, therefore this model was chosen as a suitable model to describe the release kinetics of sorafenib^54^. Similar results also were obtained for magnetic nanoparticles coated with polyvinyl alcohol.

**Figure 12:**
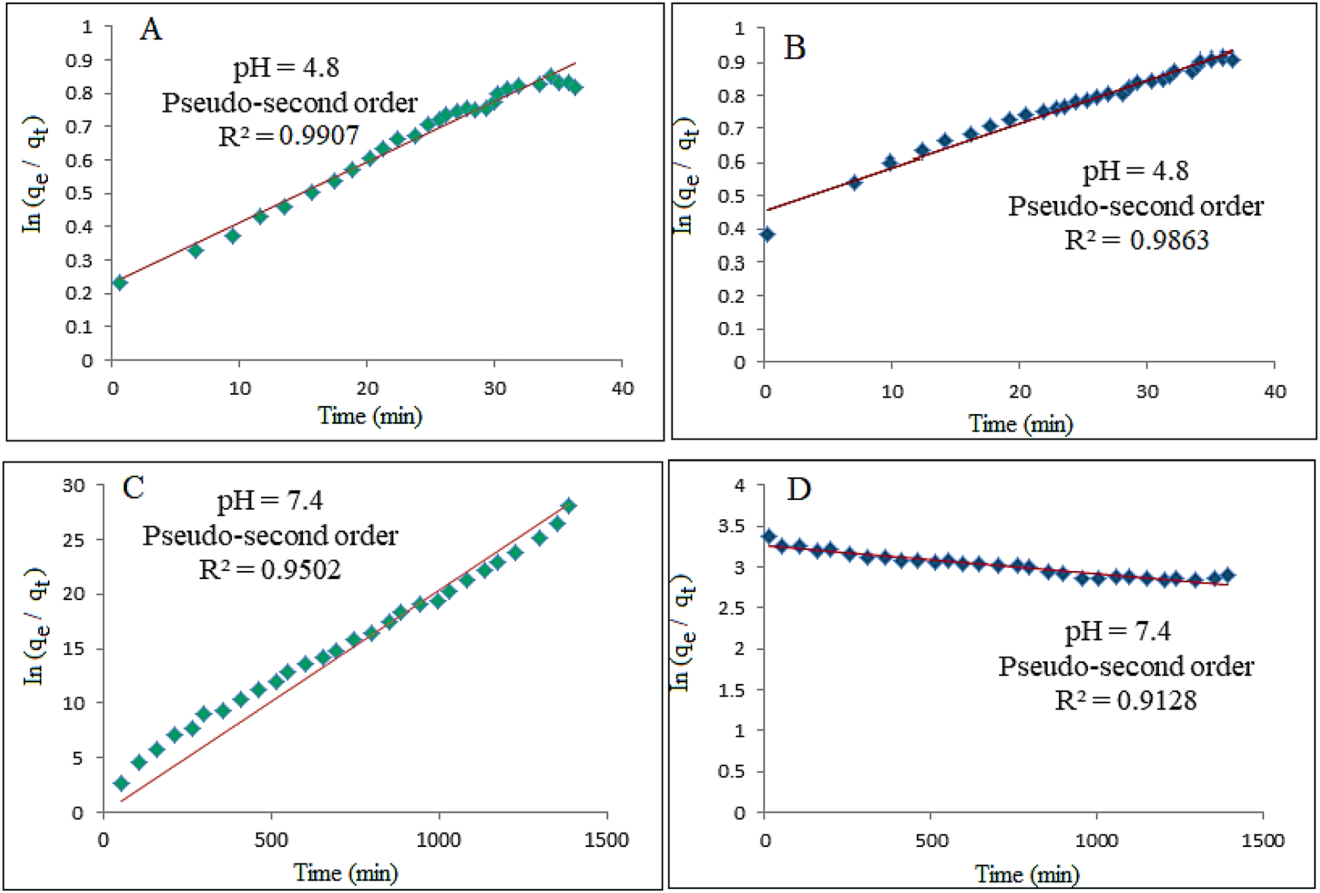
Fitting the data for sorafenib release from (**A**) FPEGSO-MLDH nanoparticles; (**B**) FPVASO-MLDH nanoparticles dissolved in dimethyl sulfoxide into different solutions to the pseudo-second-order kinetics for pH 7.4; (**C**) FPEGSO-MLDH nanoparticles; (**D**) FPVASO-MLDH nanoparticles dissolved in dimethyl sulfoxide into different solutions to the pseudo-second-order kinetics for pH 4.8. Abbreviations: t, time; q_t_, release at time t.

The results reported in Table 4 shows the half-life of the FPEGSO-MLDH and FPVASO-MLDH nanoparticles at 134 and 150 min in pH 4.8, respectively. Further, the rate constant (k) quantity for magnetic nanoparticles coated with polyethylene glycol and polyvinyl alcohol were 2.4 and 3.3 mg/min in acidic pH, with the saturation release of 99% and 98%, respectively. Using plots of the fitting of t/qt against time which is shown in Fig. 12, the correlation coefficient (R2) for the nanoparticles coated with PEG and PVA was found to be 0.99 and 0.98, respectively.

**Table 4:** The correlation coefficient, rate constant and half-life obtained by fitting the sorafenib release data into the PBS solution at pH 4.8 and pH 7.4.

Sample	pH	Saturation release (%)	R^2^			Pseudo 2nd order rate constant (k(mg/min)	t_½_
			Pseudo first order	Pseudo 2nd order	Parabolic Diffusion model		
A	4.8	99	0.9901	0.9907	0.8633	2.4 × 10^−2^	134
B	4.8	98	0.9469	0.9863	0.8552	3.29 × 10^−2^	150
C	7.4	95	0.9447	0.9502	0.8822	8.63 × 10^−5^	181
D	7.4	91	0.9009	0.9128	0.8460	7.75 × 10^−5^	213

### MTT assay

All the cytotoxicity tests were performed in triplicates and the standard deviations were incorporated in the bar graphs. The IC_50_ values were calculated by plotting x against y-axis and then the x-axis (conc.) was converted to its log values followed by nonlinear curve fitting under xy analysis to obtain a straight line in which the line equation fit as y = ax + b.

### Cytotoxicity studies on normal 3T3 fibroblast cells

**Figure 13:**
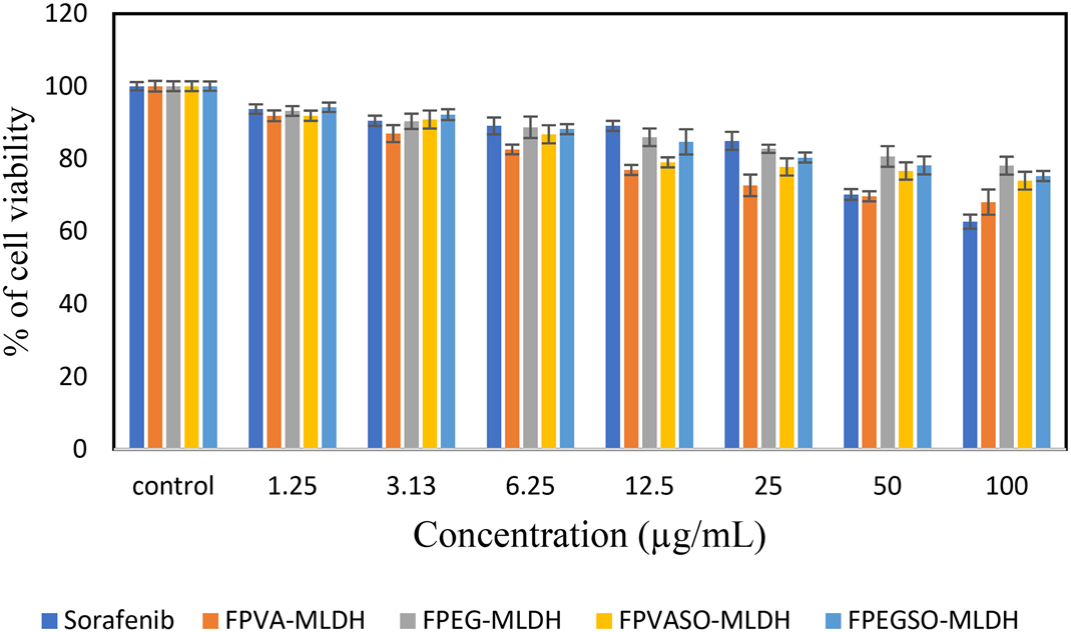
Cytotoxicity assay of Sorafenib (SO), FPEG-MLDH, FPVA-MLDH, FPEGSO-MLDH and FPVASO-MLDH against normal 3T3 cells at 72 h. Results were calculated as mean ± standard deviation for n = 3 independent experiments. * p < 0.05 compared to control (untreated cells) and treatment group using the ANOVA and Duncan’s Multiple Range Test.

The results of cytotoxicity with normal fibroblast 3T3 cells in various concentrations for FPEG-MLDH, FPVA-MLDH, FPEGSO-MLDH, and FPVASO-MLDH after 72 h is depicted in Fig. 13. According to Fig. 13, sorafenib (SO), FPEG-MLDH, FPVA-MLDH (nanocarriers), FPEGSO-MLDH, and FPVASO-MLDH were cytocompatible and did not show toxicity. This means that the formulation of anticancer nanoparticles are not harmful to normal cells and only target the cancer cells. No significance difference was observed between samples in the concentration range of 1.25–50 μg concentrations using the ANOVA and Duncan’s Multiple Range Test.

### Anticancer action against liver cancer cells, HepG2

**Figure 14:**
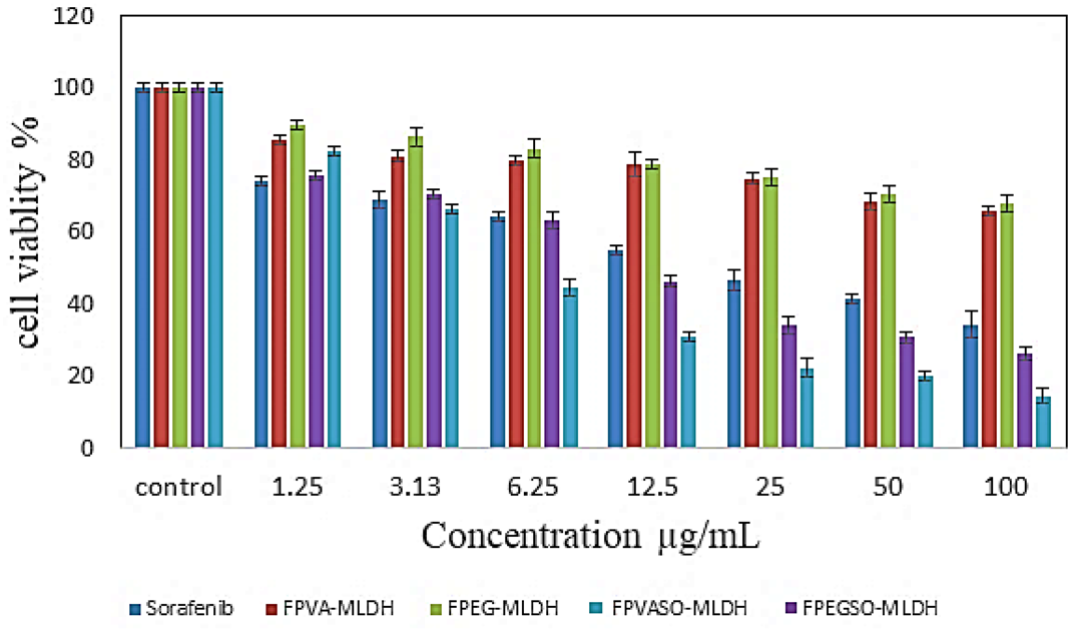
Cytotoxicity assay of sorafenib (SO), FPEG-MLDH, FPVA-MLDH, FPEGSO-MLDH and FPVASO-MLDH against HepG2 cells at 72 h of incubation. Results were calculated as mean ± standard deviation for n = 3 independent experiments. * p < 0.05 compared to control (untreated cells) and treatment group using the ANOVA and Duncan’s Multiple Range Test.

The effect of various concentrations of anticancer drug comprised of sorafenib (SO), FPEG-MLDH, FPVA-MLDH, FPEGSO-MLDH, and FPVASO-MLDH against liver cancer cells (HepG2) after incubation for 72 h is depicted in Fig. 14. There was no inhibitory effect against HepG2 cells for empty carriers; FPEG-MLDH and FPVA-MLDH at concentrations; 1.25 µg – 100 µg. The IC_50_ related to pristine sorafenib against HepG2 was 21.52 μg/mL while FPEGSO-MLDH and FPVASO-MLDH nanoparticles showed an IC_50_ of 15.15 μg/mL and 8.92 μg/mL, respectively. The sorafenib-loaded FPEGSO-MLDH and FPVASO-MLDH showed actual amounts of 69% and 54% from the percentage of the drug sorafenib in IC_50_ of the anticancer nanoparticle respectively which were determined using the HPLC analysis. The results suggested a lower IC_50_ of FPEGSO-MLDH and FPVASO-MLDH nanoparticles at a concentration of 15.15 μg/mL and 8.92 μg/mL, respectively when compared to the pristine sorafenib. Additionally, the FPEGSO-ZLDH revealed better cytotoxicity than to FPVASO-MLDH with a 69% drug loading percentage. The results for sorafenib (SO), FPEG-MLDH, FPVA-MLDH, FPEGSO-MLDH and FPVASO-MLDH shows significantly different at concentrations of 1.25 to 100 μg/mL (p < 0.05) compared to control (untreated cells). A dose-dependent anticancer effect against cancer cell line was observed for SO, FPEG-MLDH, FPVA-MLDH, FPEGSO-MLDH and FPVASO-MLDH and the IC_50_ of all the samples is summarized in Table 5. Lower IC_50_ value was observed in the formulated nanoparticles compared to free form drug is observed, indicating a potential anticancer property of the formulated nanoparticles.

**Table 5:** The half-maximal inhibitory concentration (IC_50_) value for sorafenib (SO), FPEG-MLDH, FPVA-MLDH, FPEGSO-MLDH, and FPVASO-MLDH samples tested on 3T3 and HepG2 cell lines.

Nanocomposites IC_50_ (μg/mL)	3T3 Fibroblast cell	HepG2 cells
Sorafenib (SO)	N.C	21.52
FPEG-MLDH	N.C	N.C
FPVA-MLDH	N.C	N.C
FPVASO-MLDH	N.C	8.92
FPEGSO-MLDH	N.C	15.15

Abbreviation: N.C, No toxicity at 100 µg/mL.

Overall, a comparison between 3T3 cells and HepG2 cells depicted that the nanoparticles showed significant cytotoxicity effect on the cancer cell compared to the normal cells based on the dose-dependent data. Moreover, the release of sorafenib from its nanoparticle is more efficient than the solution of pH 4.8 which mimics closely the acidic microenvironment of the cancer cells. Due to this, the nanoparticles increase the cytotoxicity of the HepG2 cells compared to the normal fibroblast cells. This makes sorafenib-loaded nanoparticles as an effective novel anticancer drug delivery system in the future.

## Conclusions

In this research work, the structural and magnetic properties of iron oxide nanoparticles prepared in the presence of different polymers after sorafenib loading were investigated by various tests and drug release experiments. The XRD study showed that the samples were composed of magnetic nanoparticles coated with a polymer, layered double hydroxide and the drug, sorafenib. In addition, the results of VSM analysis showed that for magnetic nanoparticles prepared with the two polymers, the saturation magnetization depends on the type of the polymer used, showing a lower value for the sample coated with PVA. The scanning and transmission electron microscopy images also showed that the particles became less agglomerated due to the increase in surface-to-volume ratio. Cytotoxicity studies displayed that the cytotoxicity IC50 value of the synthesized nanoparticles with polyvinyl alcohol was lower than the nanoparticles with polyethylene glycol at the same timepoint. The drug released profile favours in the nanoparticles treatment time with significantly IC50 in cell cytotoxicity. ‏Based on the results of this study, it seems that magnetic iron oxide nanoparticles coated with polymer and LDH could be suggested as a suitable drug carrier for the chemotherapy drug, sorafenib, and its application in the treatment of cancer with minimal side effects.
